# Microwave and Blanching Pretreatments for Hot Air Drying of Orange-Fleshed Sweet Potato Slices (Ipomoea batatas)

**DOI:** 10.1155/2020/8872429

**Published:** 2020-10-22

**Authors:** Ernest Abano

**Affiliations:** Department of Agricultural Engineering, School of Agriculture, College of Agricultural and Natural Sciences (CANS), University of Cape Coast, Ghana

## Abstract

Microwave and steam blanching as pretreatments to hot air drying of orange-fleshed sweet potato (OFSP) were studied. The air-drying experiment was performed at constant temperature of 70°C and airflow of 1.0 m/s. The effective moisture diffusivity varied from 1.5 × 10^−9^ to 4.4 × 10^−9^ m^2^/s, and 1.1 × 10^−10^ to 7.9×10^−10^ m^2^/s, for the microwave and blanched assisted hot air drying, respectively. The activation energy obtained for the various microwave-assisted hot air drying was 29.1 W/mm for 4 min, 68.1 W/mm for 3 min, and 79.7 W/mm for 2 min. Ascorbic acid degradation and formation of brown pigments in the OFSP slices were lower in microwave than in steam blanch-assisted drying. Microwave-assisted drying of OFSP is best governed by Page model, *MR* = exp(−k*t*^n^), while the blanch-assisted followed the logarithmic model, *MR* = *a* exp(−k*t*) + c. To produce better quality OFSP flour, it is recommended to cut the tubers into 3 mm slices, microwave at a power of 630 W for 2 min or blanch for 1 min, 43 seconds prior to hot air drying.

## 1. Introduction

Ghana is the third poorest country in the world with severe vitamin A deficiency (VAD) problems. The prevalence of VAD in Ghana is estimated at 76% [1] [2], which is close to double that of Africa's average of 41.9%. VAD prevalence is the highest among children under 5 years of age and among women of childbearing age, which has worsened from an estimated amount of 20% in 2004 to its current rate of 35.6% [3]. The white-fleshed sweet potato is traditionally the most consumed sweet potato varieties among Ghanaians, as a carbohydrate source. Through an integrated agricultural and nutrition intervention biofortification programmes, the orange-fleshed sweet potato (OFSP) was introduced with the sole aim of combating the VAD problems in Ghana and many African countries. Orange-fleshed sweet potatoes, OFSP (Ipomoea batatas), are among the best-known sources of provitamin A carotenoids, with about 85% of the carotenoids capable of been absorbed and converted into vitamin A in the human body [4] [5]. However, there are unsold orange-fleshed sweet potato roots by farmers who produce them especially during bumper harvest, because consumers complain of its undesirable soft texture after boiling, rendering boiled OFSP unappealing, making the produce go waste and unavailable year-round. Farmers' livelihoods are seriously affected due to the unsold commodities. In addition, there is unsatisfied demand by farmers and processors to possess adequate knowledge and skills to process OFSP into secondary and tertiary products or develop it into OSFP-containing products. Drying is among the best-known technologies used to produce quality products, which can be used as ingredients for the preparation of bread, banku, kenkey, and yoghurt, and to reduce postharvest losses associated with OFSP [6]. In addition, the carotenoid in the OFSP is heat sensitive, and as a result, an appropriate method is required to preserve the orange color to produce high-quality products. Microwave and steam blanching prior to drying have been used extensively in the food industry [6, 7]. Blanching is the most commonly used method of pretreatment in the food industry due to its ease of use and efficacy in the deactivation of pathogenic microorganisms, enzymes, and the retention of food color and aroma. Many research evidences in favour of microwave-assisted drying of fruits and vegetables and root and tubers have been provided [6, 8–10]. The objective of microwave drying is to speed up drying process, increase mass transfer, and produce good quality products, while blanching prior to processing fruits and vegetables accelerates drying rate, prevents color changes, softens the texture, denatures enzymes, and destroys contaminating microorganisms. Microwave blanching is caused by the rotation and collision of water molecules caused by the variation of the electromagnetic field generating heat [11]. In conventional blanching, the material internal temperature distribution depends on the geometric shape and its thermal conductivity. Microwave blanching results in the instantaneous heating of all the individual elements of a material. Microwave blanching time can be significantly reduced as compared to the conventional blanching methods [12]. Microwave blanching has advantages over the conventional blanching methods: uniformity of heating, low energy consumption, and high retention of bioactive compounds [13]. During drying, the root needs to be sliced into appropriate thickness to facilitate the drying process. Therefore, the objective of the present study was to investigate the effect of microwave and steam blanching pretreatment prior to air drying kinetics and quality attributes such as beta-carotene, ascorbic acid, and nonenzymatic browning of different slices of dried OFSP.

## 2. Materials and Method

### 2.1. Sample Preparation

The orange-fleshed sweet potato (OFSP) roots were obtained from an accredited farmer by the Ministry of Food and Agriculture (MOFA), Ghana, at its commercial maturity and brought to the laboratory. The roots were cleaned; sliced into 3, 6, and 9 mm thicknesses; soaked in 5% citric acid and 1% sodium benzoate solution for 10 min; and kept in the freezer at a temperature of -18C. This was done to avoid excessive discolouration and enzymatic browning of the samples prior to drying. The initial moisture content was determined by drying 10 g of the sample in triplicate in a hot air oven at a temperature of 105°C for 24 hours [14].

### 2.2. Experimental Design

A response surface method involving 2 factors and 3-level factorial design using Minitab version 17 with 3 replications was used to design the drying experiments. The effect of two factors: microwave power denoted as *X*_1_ (385 W–697 W) and microwave time denoted as *X*_2_ (2 min-4 min). The dependent variables are (ascorbic acid, beta-carotene, browning index, and drying time).

### 2.3. Microwave and Blanching Pretreatment

A domestic microwave (Daewoo KOR-6 L77) with a frequency of (2450 MHz) was used to pretreat OFSP slices (10 mm) for 2, 3, and 4 minutes. Blanching was done using the steam from the boiling water by placing the sliced OFSP samples in a perforated stainless-steel bowl 10 cm from the source of the boiling water. Fifty (50) grams sample of uniform geometry was placed in a microwave for 2, 3, and 4 minutes or blanched for 1, 2, and 3 min. After blanching, the excess moisture at the surface of the sample was blotted with absorbent paper and subjected to hot air drying.

### 2.4. Hot Air Drying

The pretreated OFSP slices were dried at a temperature of 70°C on a meshed sample holder with a cabinet dryer (Model: PT-40,220-240 V, 50/60 Hz, China). The oven was run idle for 1 h prior to the drying experiment to achieve steady-state conditions. 50 g pretreated samples were used for each drying experiment. During drying, the moisture content of the slices was monitored every 30 min at the initial stages and later changed to 1 hr until the moisture content reached 8% (dry basis). The time taken for the sample moisture content to reach 8% was used to indicate the drying time (DT).

### 2.5. Determination of Ascorbic Acid

Ascorbic acid was measured according to with slight modification (Kapur, Haskovic, Klepo, &) [15]. One (1) gram of the sample was homogenized with 5 ml metaphosphoric-acetic acid solution in a total volume of 10 ml. The solution was filtered and centrifuged at 3000 rpm for 20 min, after which the supernatant was used for spectrophotometric determination. A 1.54 ml of the supernatant, 90 *μ*l of bromine water, 50 *μ*l of 10% thiourea, and 390 *μ*l of 2,4-dinitrophenylhydrazine solution was added and incubated at 37°C for 3 hours. After the incubation, the samples were cooled in an ice bath for 30 min. A 1.92 ml of chilled 85% H_2_SO_4_ was added with constant stirring. A blank was also prepared and its absorbance measured at 521 nm using a spectrophotometer. A graph of ascorbic acid concentration versus absorbance at 521 nm was plotted to calculate the ascorbic acid content in the sample.

### 2.6. Nonenzymatic Browning Determination

The nonenzymatic browning index (BI) was determined as reported in previous study [6].

### 2.7. Drying Kinetics

The drying kinetics of OFSP was expressed in terms of empirical models, where the experimental data obtained was plotted in the form of a dimensionless moisture ratio (MR) against drying time in minutes. The MR of the OFSP was determined using Eq. (1).

(1) $$\begin{array}{c} \text{MR}=\left(\frac{M-M_{e}}{M_{O}-M_{e}}\right) \end{array}$$

Where MR is the moisture ratio, *M*_*O*_ is the initial moisture content (g water/g dry matter), *M* is the moisture content at any time (g water/g dry matter), and *M*_*e*_ is the equilibrium moisture content (g water/g dry matter) ([16]).

Three empirical drying models widely used in scientific literature: Page, Henderson and Pabis, and Logarithmic were fitted to the experimental data set (MR, *t*) shown in Table 1 to describe the drying kinetics of OFSP.

The algorithm for the estimation of the model fitting was performed using the modified Levenberg-Marquardt algorithm [17]. Given an initial value for the constant in the model, the objective function is solved. For *k* + 1, *k* = 0, 1, 2, ⋯, the function *f*(*k*)*i* = *fi*(∅(*F*)), *R*(*k*)*i* = *yi* − *f*(*k*)*i*, *Fk* = *F*(∅(*k*)), and *J*(*k*) = *J*(∅(*k*)) was computed. A positive scalar was chosen such that *F*(∅(*k*) + *hk*) < *Fk* where *hk* = −(*J*(*k*)′*J*(*k*) + *αkI*) − 1*J*(*k*)^"^*R*(*k*). The function ∅(*k* + 1) = ∅(*k*) + *hk* and *J*(*k* + 1),  *R*(*k* + 1),  *W*(*k* + 1), and *Fk* + 1  was computed. If any of 1 − (*Fk* − 1/*Fk*) < *ε*1(*SSCON*); |*hki*/∅(*k*)*i*| < *ε*2(*PCON*); *k* + 1 ≥ ITER_max_; |*r*(*k* + 1)*j*| < *ε*2(RCON); where *r*(*k* + 1)*j* is the correlation between the jth column *J*(*k* + 1) and *W*(*k* + 1)*R*(*k* + 1) was satisfied then the algorithm stops and ∅∗ = ∅(*k* + 1) is predicted and the stop reason is stated else, the iteration continues.

The reduced chi-square (*χ*^2^), root mean square error (RMSE), and the coefficient of determination (*R*^2^) (Eqs. (2), (3), and (4)), respectively, were used as the primary criteria to select the best model [18].

(2) $$\begin{array}{c} \chi^{2}\frac{\sum_{i=1}^{N}\left({\text{MR}}_{\text{expt},i}-{\text{MR}}_{\text{pred},i}\right)2}{N-z}, \end{array}$$

(3) $$\begin{array}{c} \text{RMSE}=\sqrt{\frac{1}{N}\overset{N}{\underset{i=1}{\sum }}{\left({\text{MR}}_{\text{expt},i}-{\text{MR}}_{\text{pred},i}\right)}^{2}}, \end{array}$$

(4) $$\begin{array}{c} R^{2}=\frac{N\sum_{i=1}^{N}{\text{MR}}_{pred,i}{\text{MR}}_{expt,i}-\sum_{i=1}^{N}{\text{MR}}_{pred,i}\sum_{i=1}^{N}{\text{MR}}_{expt\;x,i}\;}{\sqrt{\left[N\sum_{i=1}^{N}\text{M}{{\text{R}}^{2}}_{pred,i}-{\left(\sum_{i=1}^{N}{\text{MR}}_{pred,i}\right)}^{2}\right]\;\left[N\sum_{i=1}^{N}{{\text{MR}}^{2}}_{expt,i}-{\left(\sum_{i=1}^{N}{\text{MR}}_{expt,i}\right)}^{2}\right]}}. \end{array}$$

Where MR_exp,*i*_ is the experimental moisture ratio, MR_pred,*i*_ is the predicted moisture ratio, *N* is the number of observations, *z* is the number of constants in the drying model. Based on the criteria of the lowest reduced chi-square and RMSE and the highest *R*^2^, the best model describing the thin layer drying characteristics was chosen. The drying rate constants and coefficients of the model equations were determined with nonlinear regression of SPSS 23.0, and the goodness of fit of the curves was determined with correlation analysis.

### 2.8. Determination of Moisture Diffusivity

Fick's second law of diffusion, which characterizes moisture migration during thin-layer drying of food materials, was used to calculate the effective moisture diffusivity, considering a constant moisture diffusivity, infinite slab geometry, and uniform initial moisture distribution (Crank, 1975). During drying, it is assumed that diffusivity explained with Fick's diffusion equation is the only physical mechanism to transfer the water to the surface of the materials to be dried (Dadalı, Kılıç Apar, & Özbek, 2007).

(5) $$\begin{array}{c} \text{MR}=\frac{8}{\pi^{2}}\overset{\infty }{\underset{n=0}{\sum }}\frac{1}{{\left(2n+1\right)}^{²}}\;\exp\left[-\frac{{\left(2n+1\right)}^{2}\pi^{2}D_{\text{eff}}\;t}{4L^{2}}\right] \end{array}$$

Where *D*_eff_ is the effective diffusivity (m^2^/s) and *L* is half the thickness of slice of the sample (*m*). Equation (5) can be simplified to the following for long drying times.

(6) $$\begin{array}{c} \text{MR}=\frac{8}{\pi^{2}}\;\exp\left[-\frac{\pi^{2}D_{\text{eff}}\;t}{4L^{2}}\right]. \end{array}$$

*D*_eff_ of the OFSP slices was obtained from the slope (*K*) of the graph of InMR against the drying time. InMR versus drying time results in a straight line with negative slope, and *K* is related to *D*_eff_ by [Eq. (7)]

(7) $$\begin{array}{c} K=\frac{\pi^{2}D_{\text{eff}}\;t}{4L^{2}}. \end{array}$$

*D*_eff_ can be related to the temperature by Arrhenius (Equation (8))

(8) $$\begin{array}{c} D_{\text{eff}}=D_{0}\exp\left[-\frac{E_{a}}{R\left(T+273.15\right)}\right]. \end{array}$$

Where *D*_0_ is the constant Arrhenius equation (m^2^/s), *E*_*a*_ is the activation energy (kJ/mol), *T* is the temperature of hot-air (°C), and *R* is the universal gas constant (8.31451 kJ mol^−1^ K^−1^). Equation (8) can be rearranged into the form:

(9) $$\begin{array}{c} \text{In}\left(D_{\text{eff}}\right)=\text{In}\left(D_{0}\right)\left[-\frac{E_{a}}{R\left(T+273.15\right)}\right]. \end{array}$$

The activation energy for moisture diffusion was obtained from the slope of the graph of In(*D*_eff_) against -1/R(T+273.15) (Kabiru, Joshua, & Raji, 2013).

### 2.9. Calculation of Activation Energy

According to Pillai and Green (2010), for the standard microwave oven drying procedure, the internal temperature of sample is not an assessable variable. Therefore, the use of Arrhenius-type equation is considered for illustrating the relationship between the diffusivity coefficient and the ratio of the microwave power output to sample thickness instead of temperature for the calculation of the activation energy. The activation energy is found as modified from the revised Arrhenius. The equation as suggested by (Dadalı et al., 2007) is represented as

(10) $$\begin{array}{c} D_{\text{eff}}=D_{o}\;\exp\left[\frac{E_{a}q}{P}\right]. \end{array}$$

Where *D*_*o*_ is the constant in the Arrhenius equation (m^2^/s), *E*_*a*_ is the activation energy (W/mm), *P* is the microwave power (W), and *q* is the sample thickness (mm). Eq.(10) can be rearranged as

(11) $$\begin{array}{c} \text{In }\left(D_{\text{eff}}\right)=\text{In }\left(D_{o}\right)-\frac{E_{a}q}{P}. \end{array}$$

The activation energy for moisture diffusion was obtained from the graph of ln (*D*_eff_) against *q*/*P*.

### 2.10. Optimization of the Drying Process

The optimization of the drying process was performed using a response optimizer techniques in Minitab herein referred to as composite desirability index, CDI (Myers & Montgomery, 2001) using Eq. (12).

(12) $$\begin{array}{c} \text{CDI}={\left[\overset{n}{\underset{i=1}{\prod }}di\left(Y_{i}\right)\right]}^{1/n}. \end{array}$$

The *di* represents the desirability index for each response variable (*Y*_*i*_), and *n* is the number of response variables. The CDI ranges between 0 and 1 with 0 being the least desired whereas 1 is the most desirable. Maximization of DI value is the goal in optimization studies. The optimization process incorporates goals and target for the factors and the responses. Regarding this study, minimization of drying time (DT) and browning index (BI) as well as maximization of ascorbic acid (AA) content were desired while maintaining the factors at any level within the design values.

### 2.11. Statistical Analysis

ANOVA was performed at a probability of 95% confidence interval to reveal the statistical significance of the model term. The accuracy of the model to describe the response variables was diagnosed against the normal probability plots of the residuals, the predicted versus actual plots, and the coefficients of determination (*R*^2^) values. The 3D surface plots for the factors were generated for the various response.

## 3. Results and Discussion

### 3.1. Microwave and Blanch Assisted Drying Kinetics

**Figure 1:**
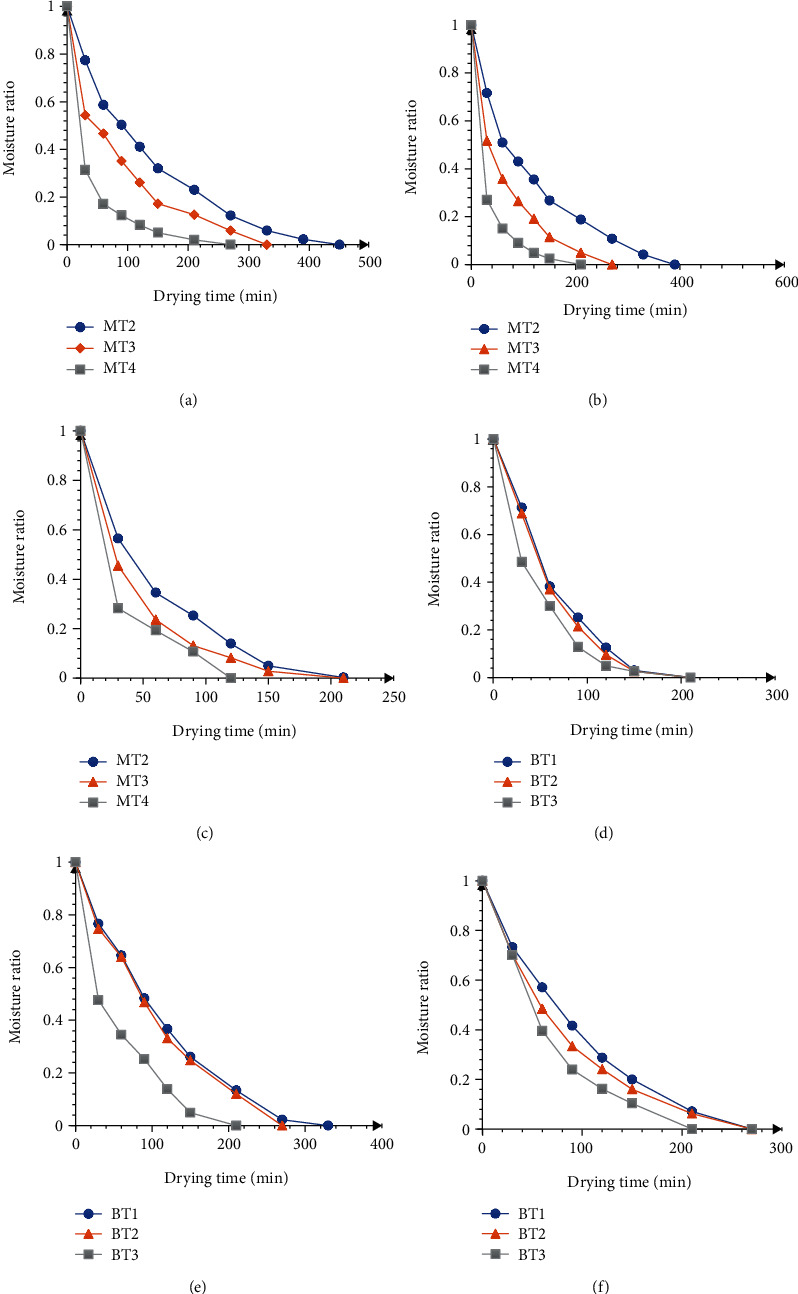
Variation of moisture ratio versus drying time for microwave-assisted drying (a) 385 W, (b) 541 W, and (c) 697 W and blanch-assisted drying (d) 3 mm, (e) 6 mm, and (f) 9 mm at 70°C.

**Figure 2:**
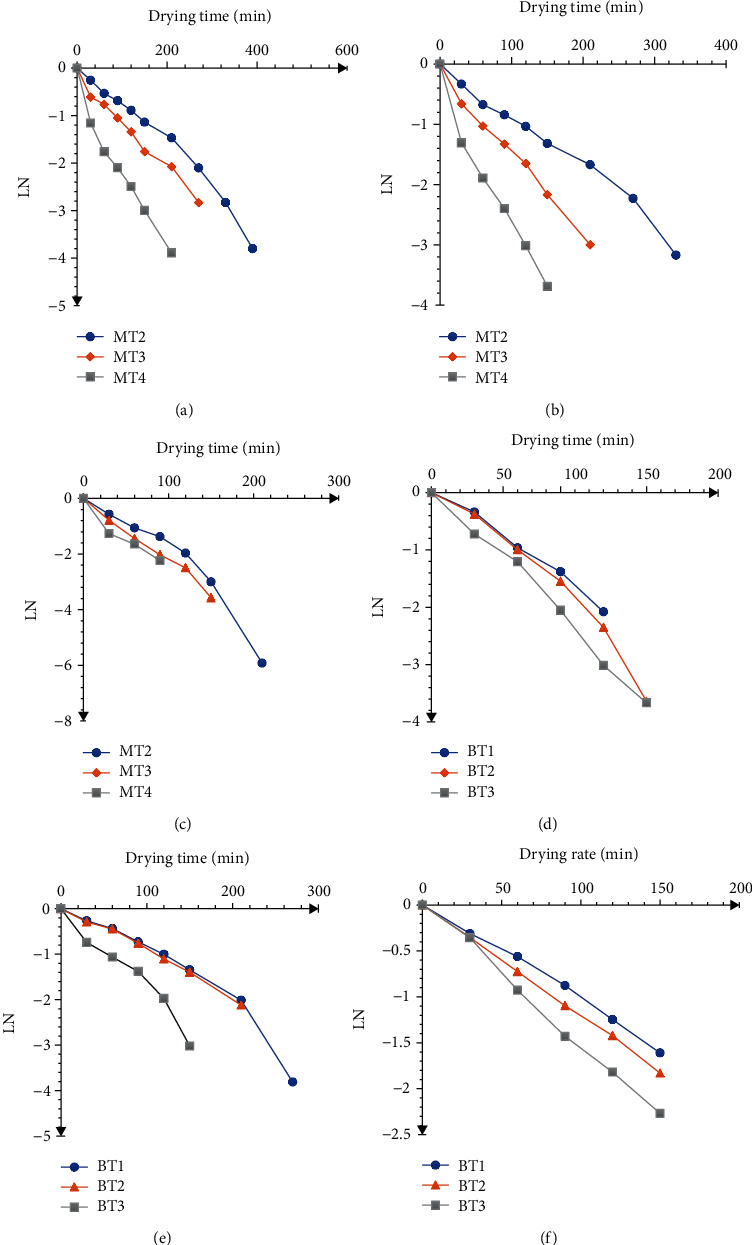
Variation of ln (MR) against drying time plots for Variation of ln (MR) against drying time plots for microwave-assisted hot air drying at (a) 385 W, (b) 541 W, and (c) 697 W blanched-assisted drying samples with thickness (d) 3 mm, (e) 6 mm, and (f) 9 mm at 70°C. MT2, MT3, and MT4 are 2 min, 3 min, and 4 min microwave treatments. BT1, BT2, and BT3 are 1 min, 2 min, and 3 min blanching treatments, respectively.

Fresh OFSP with an initial moisture content (86% wet basis) were dried until their equilibrium moisture content was reached in a hot air cabinet dryer. The drying curves of OFSP pretreated at various microwave powers (385, 541, and 697 W) and times (1, 2, and 3 min) prior to hot air drying at 70°C are shown in Figure 1. The product moisture content decreased with drying time. Variation in moisture content within the product causes moisture gradients to remove moisture from the samples. Generally, as microwave power increases from 385 W to 697 W, the moisture gradient increased resulting in decreased drying time. Increased porous structure formation in the tissues of the sweet potato as a result of increased exposure by the electromagnetic waves from the microwave increased the liquid diffusion rate. Both pretreatment prior to drying took place in a falling rate period (Figure 1), showing that moisture removal from the OFSP slices was driven primarily by molecular diffusion. The results agree with drying of potato slices by (Akpinar, Midilli, & Bicer, 2003). Contrary to this, steam blanching decreased the rate of moisture removal leading to an increase in drying time, as shown in Figure 2. Similar findings have been reported ([6]; Leng, Gouado, & Ndjouenkeu, 2011; Quansah, Saalia, Abbey, & Annor, 2010). When the OFSP slices are blanched, the cells may become less permeable to moisture transfer, as there is an increase in starch gelatinize [6].

### 3.2. Pretreatments Effect on Moisture Diffusivity and Activation Energy

**Figure 3:**
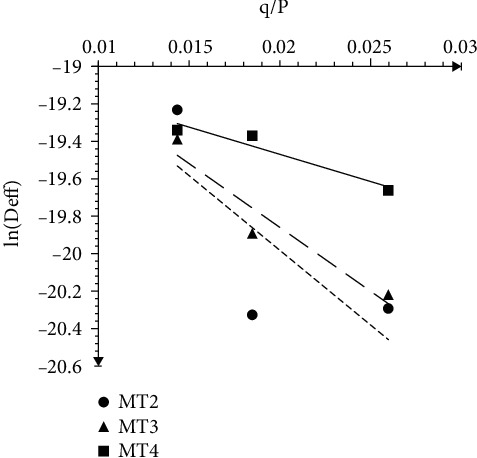
Variation of In *D*_eff_ against *q*/*P* for microwave-assisted air drying.

The constant *D*_eff_ in Fick's law of diffusion is the product mass diffusivity, which measures how quickly moisture diffuses through food materials. The effect of microwave and steam blanching pretreatments on the effective moisture diffusivity (*D*_eff_) of OFSP is shown in Figure 2. The *D*_eff_ values for the microwave-assisted and blanch-assisted hot air drying at 70°C are presented in Table 2. The *D*_eff_ values ranged from 1.5 × 10^−9^ to 4.4 × 10^−9^ m^2^/s for microwave-assisted air drying and 1.1 × 10^−10^ to 7.9 × 10^−10^ m^2^/s for blanch-assisted air drying. These values lie within the general range of 10^−12^–10^−8^ m^2^ s^−1^ for drying of food materials [20]. It is clear from Table 2 that the effective moisture diffusivity for microwave-assisted air drying is by far higher than its blanch-assisted counterparts. Generally, the *D*_eff_ values increased with an increase in microwave power and exposure time. The increased *D*_eff_ as microwave power and exposure times increase has been attributed to increased heating energy, which increases the activity of the water molecules in the test samples (Mahdi, Ali, & Mohammad, 2013). The results agree with microwave-vacuum drying of tomato slices [19]. The *D*_eff_ of the blanch-assisted samples on the other hand did not follow a similar trend as it did with microwave-assisted air drying. At 6 mm and 9 mm sample thicknesses, the increase in blanching time did not necessarily lead to a significant increase in effective moisture diffusivity (Figure 3). At sample thickness of 6 mm, the effective moisture diffusivity decreased with an increase in blanching time, while for the 9 mm thick samples, the *D*_eff_ increased with blanching time. Case-hardening as sample thickness increases up to some point has been blamed for such fluctuations in effective moisture diffusivity. This supports the theory that moisture diffusivity is a complex and system specific function. The internal mass transport property, which characterizes the *D*_eff_, from food materials is driven by molecular diffusion, liquid diffusion, vapour diffusion, hydrodynamic flow, and other possible mass transport mechanisms [21].

The activation energy values obtained from the variation of In*D*_eff_ against *q*/*P* for microwave-assisted air drying (Figure 3) were 29.1 W/mm for 4 min, 68.1 W/mm for 3 min, and 79.7 W/mm for 2 min. It is evident that the microwave power-dependent activation energy for moisture removal in the OFSP slices increased with pretreatment time. However, the temperature-dependent activation energy obtained for the blanch-assisted hot air could not be estimated, since temperature was not varied in this study. The activation energy values were within the general range of 12.7-110 kJ/mol (Aghbashlo & Samimi-Akhijahani, 2008) for most agricultural and food materials as presented by several other reports. The value observed in this study is comparable to 46.9 W/mm for tomato samples [19].

### 3.3. Modelling of the Drying Curves

Nonlinear regression analysis was used to determine the appropriate mathematical model for drying OFSP. The dimensionless moisture ratio was plotted against drying time for the experimental data at various pretreatment conditions. The data was fitted to three drying models commonly used in drying literature: Page, Henderson and Pabis, and Logarithm models. The best model describing the thin layer drying characteristics of OFSP during microwave drying was chosen as the one with the lowest chi square (*χ*^2^), root mean square error (RMSE) and the highest correlation coefficient (*R*^2^). The results of the experimental data for the samples dried using microwave and blanch-assisted hot air drying at various microwave powers of 385, 541, and 697 W are shown in Tables 3 and 4, respectively.

The results obtained from the microwave and blanch-assisted air drying at 70°C indicated that the *R*^2^ value for the three models were all above 0.92. The statistical parameter estimations showed that the RMSE and *χ*^2^ values ranged from 1E-05 to 0.0408 and 2E-07 to 0.0017, respectively, showing the all the models tested could be adequately used to predict microwave and blanch-assisted drying of orange fleshed sweet potato slices. The relatively high values of correlation coefficients, low reduced chi-square, and low root mean square errors indicate a good predicting capacity for the two pretreatments over the entire duration of the drying process. Among the models tested, the Page model was the most appropriate for the microwave-aided air drying of OFSP, since it had the highest value for the coefficient of determination (*R*^2^) and the lowest reduced chi-square (*χ*^2^) and root mean square error (RMSE). The Logarithmic model on the other hand provided the best agreement between the experimental and predicted moisture ratio values for the blanched-assisted air drying of OFSP at 70°C. It was observed in Tables 3 and 4 that the values of drying rate constant (*k*) predominantly increased with increase in microwave power, pretreatment time, and blanching time. This implies that drying rate potential of OFSP generally increased with an increase in microwave power and pretreatments time for both methods. However, between 541 W and 697 W, the drying rate constant decreased for all pretreatments times. This suggests that the most critical microwave power for optimum removal of moisture lie within this range for microwave drying of OFSP. As the OFSP thickness increased from 3 mm to 6 mm, the drying rate potential reduced and subsequently increased when the sample thickness increased to 9 mm. This phenomenon is consistent with microwave and blanch assisted drying for dioscorea rodundata or white yam [6].

### 3.4. Microwave and Blanch-Assisted Drying on Drying Time

**Figure 4:**
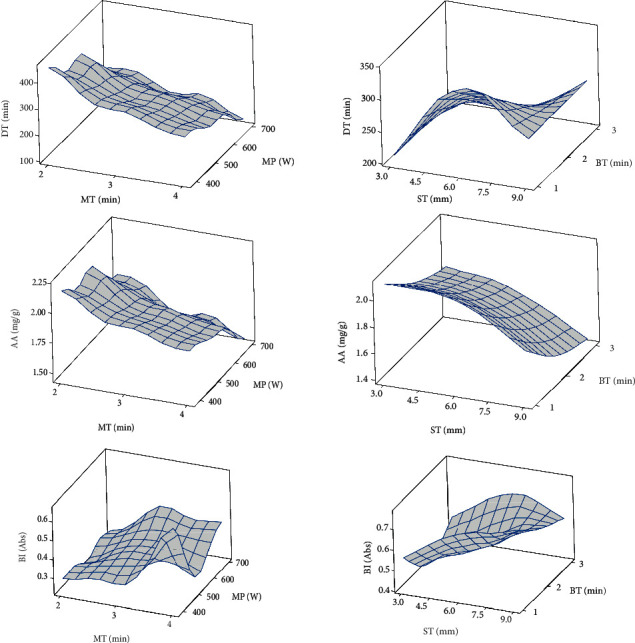
Effect of microwave and blanching pretreatments on drying time (DT), ascorbic acid content (AA), and browning index (BI).

The effect of microwave and blanching assisted drying on drying time of OFSP slices is displayed in Table 5. Both microwave power and their exposure time were significant (*P* < 0.005) for the main, interaction as well the curvature regions of the model (Table 6). Increase in microwave power and exposure time decreased the drying time significantly (*P* < 0.05). Drying time decreased from 420 min to 120 min as power increased from 385 W to 697 W. Meanwhile, the relative contribution of microwave power and exposure time was different. The increase in the intensity of the electromagnetic waves from 385 to 697 W had a profound positive influence on the drying time than its exposure time. This is because of the higher value of the microwave power coefficient compared with its exposure time (Table 6). The combined effect of microwave intensity and its exposure time was positive on the drying time (Figure 4). This was contrary to the observations of the blanch-assisted air drying of OFSP. Both the blanching time and sample thickness did not significantly impact the drying time of the samples; although, an increase in blanching time from 1 to 3 mins negatively affected it. Similar to microwave power, the sample thickness had a profound influence on drying time than blanching time. Drying time in microwave-assisted drying was faster than in blanching indicating that microwave-assisted drying reduced the entire drying process hence resulting in a substantial savings in drying time. Many researchers have reported similar findings with microwave drying for yam, okra, and cherry tomatoes ([6]; Doymaz, 2005; Muratore, Rizzo, Licciardello, & Maccarone, 2008).

### 3.5. Microwave and Blanch-Assisted Air-Drying Effect on Ascorbic Acid

The results of the experiment show that microwave power and its exposure time had significant effect on the ascorbic acid (AA) content of the dried OFSP slices (Table 6). As the microwave power increased from 385 to 697 W, there was a reduction in AA of the OFSP slices due to the destruction of the vitamin C by the electromagnetic waves. Abano and Amoah [6] had reported losses of ascorbic acid during microwave-assisted drying of white yam cubes. Also, Zheng and Lu (2011) reported a reduction of ascorbic acid with microwave drying of green asparagus. Similarly, both blanching time and its combined blanching time-sample thickness increment similarly reduced the AA content of dried OFSP slices (Figure 4 and Table 6). Significant losses of ascorbic acid in red pepper and potato due to water blanching and subsequent subjection to dehydration have been reported [22, 23]. Nevertheless, steam and microwave blanching are noted to retain a higher amount of ascorbic acid [6, 23]. Ascorbic acid is heat sensitive and very reactive and degrades mainly by thermal or oxidative means. Oxidative degradation of ascorbic acid mainly occurs due to long drying times arising from increased sample thickness and leaching out from excessive duration of blanching may have caused such decreases in ascorbic acid. Leng et al. (2011) reported more than 50% ascorbic acid losses during the blanching of *Dioscorea schimperiana*. The current study revealed that retention of ascorbic acid by microwave pretreatment is higher than by steam blanching.

### 3.6. Microwave and Blanch-Assisted Air-Drying Effect on Browning Index

Browning is a quality indicator in dried agricultural products and is caused mainly by enzymes, often called enzymatic browning, and/or heat application usually termed nonenzymatic browning. Browning is not a desirable indicator in dried OFSP and some food processing units. It is the main issue with minimally processed sweet potatoes, and so in this study, the OFSP slices were soaked in 5% citric acid and 1% sodium benzoate solution prior to blanching by microwave and steam. Therefore, the reason for these pretreatments prior to air drying was to inactivate the lipoxygenase and polyphenol oxidase enzyme responsible for enzymatic browning and color changes in fruits and vegetables [24]. Microwave-assisted air drying did not significantly affect the browning index of the OFSP but blanching time did. Browning index of microwave-assisted air-dried OFSP ranged from 0.236 to 0.657 Abs unit, while that of blanch-assisted air-dried samples ranged from 0.415 to 0.774 Abs unit. This means that in terms of brown pigment formation, the rate was slower in the microwave-assisted dried samples than the blanched samples (Figure 4), which made microwave application prior to hot air drying a better technology to produce higher quality dried product. Increase in both microwave power and steam blanching time reduced the brown pigment formation although not significant in the factor range employed. In this study, browning caused by enzymes was assumed to be negligible due to the extent of pretreatments given to the OSFP samples. Hence, the browning index determined was more of thermal effect or nonenzymatic. Plausibly, nonenzymatic browning is mainly caused by the reaction between the nitrogenous compounds and reducing sugars, nitrogenous compounds and organic acids, and sugars and organic acids owing to heat application (Cernîşev, 2010). OFSP is rich in sugars, including reducing sugars such as glucose and fructose and nonreducing disaccharide sucrose and reducing disaccharides maltose, which when subjected to heat caused browning. Lower thermal intensity and shorter drying times resulting from microwave pretreatments may have caused low reaction rate to form fewer brown pigments than in the steam-blanched OFSP slices. Namtip et al. (2006) blanched and dried sweet potato chips at 70°C–90°C and observed that the rate of browning increased with temperature from 0.039 to 0.755 absorbance units. Abano and Amoah [6] studied microwave- and blanch-assisted drying of white yam and reported similar trends; although, the brown color formation from microwave was far lower (0.035 to 0.112 Abs units).

### 3.7. Optimization of the Drying Parameters

The affirmation of the location of the optimal microwave- and blanch-assisted air-drying conditions was conducted using the concept of the composite desirability index in Minitab version 17. The predicted DT, AA, and BI were 215 min, 1.971 mg/g, and 0.262 Abs unit, respectively, for microwave-assisted air drying at 70C. For blanched-aided drying, the predicted DT, AA, and BI, were 307 min, 1.95 mg/g, and 0.433 Abs unit. The results predicted with 95% confidence in the range of factors gave optimal microwave power of 630 W and pretreatment time of 2 min for microwave-assisted drying and optimal blanching time of 1 min and 43 seconds and a sample thickness of 3 mm.

## 4. Conclusion

Microwave- and blanch-assisted air drying was carried out on orange-fleshed sweet potato (OFSP). Effective moisture diffusivity was higher for microwave than in blanch-assisted air drying. The intensity of the electromagnetic wave had a profound positive influence on the drying time than its exposure time. Drying rate potential was significantly higher in microwave than with blanch-assisted drying for the all the drying models tested. The page model best described microwave-assisted drying of OFSF, while the Logarithmic model was best for blanch-assisted drying. The quality attributes of the products in terms of ascorbic acid retention and brown pigment formation for the dried orange-fleshed potato with microwave were better than blanched-assisted drying. The study has shown that microwave blanching could be used to produce better quality OFSP flour with enhanced moisture diffusivity, and minimization of browning and ascorbic acid than conventional steam blanching.

## Figures and Tables

**Table 1:** Mathematical models that were applied to the experimental data.

Model name	Model expression	References
Page	MR = exp (−*kt*^*n*^)	[19]
Henderson and Pabis	MR = a exp(−*kt*)	[16]
Logarithm	MR = aexp(−*kt*) + *c*	[20]

**Table 2:** Moisture diffusivity for microwave and blanch assisted air drying at 70°C and their *R*^2^ values.

Microwave-assisted				Blanch-assisted			
MP (W)	MT (min)	*D*_eff×10^−9^_ (m^2^/s)	*R* ^2^	ST (mm)	BT (min)	*D*_eff×10^−10^_ (m^2^/s)	*R* ^2^
385	2	1.5	0.973	3	1	2.6	0.988
	3	1.7	0.984		2	3.6	0.958
	4	2.9	0.962		3	3.8	0.992

541	2	1.5	0.981	6	1	7.9	0.921
	3	2.3	0.991		2	6.1	0.990
	4	3.9	0.972		3	1.1	0.957

697	2	4.4	0.913	9	1	1.4	0.995
	3	3.8	0.988		2	1.7	0.997
	4	4.1	0.935		3	2.1	0.997

**Table 3:** Parameters and statistical results for the various drying models for microwave-assisted drying at 70°C.

Microwave power		385 W				541 W				697 W			
Model name	PT (min)	Model constants	*R* ^2^	RMSE	*χ* ^2^	Model constants	*R* ^2^	RMSE	*χ* ^2^	Model constants	*R* ^2^	RMSE	*χ* ^2^
Page	2	k: 0.009 n: 0.976	0.995	0.0236	6E-04	k: 0.017 n: 0.870	0.994	0.025	6E-04	k: 0.022 n: 0.946	0.995	0.0283	0.0008
	3	k: 0.040 n: 0.747	0.989	0.0333	1.1E-03	k: 0.047 n: 0.758	0.995	0.0224	5E-04	k: 0.040 n: 0.878	0.999	0.000	0.000
	4	k: 0.157 n: 0.585	0.999	0.0129	2E-04	k: 0.164 n: 0.606	0.999	0.0141	2E-04	k: 0.138 n: 0.636	0.992	0.0408	0.0017

Henderson and Pabis	2	k: 0.007 a: 0.987	0.995	0.0005	3E-07	k: 0.009 a: 0.965	0.990	0.0013	1.3E-06	k: 0.017 a: 0.990	0.994	0.0008	6E-07
	3	k: 0.012 a: 0.935	0.971	0.0031	9.9E-06	k: 0.016 a: 0.960	0.983	0.0022	4.7E-06	k: 0.024 a: 0.991	0.997	0.0004	2E-07
	4	k: 0.032 a: 0.983	0.98	0.0025	6.3E-07	k: 0.037 a: 0.990	0.987	0.002	4E-06	k: 0.034 a: 0.987	0.978	0.0046	2.2E-05

Logarithm	2	k: 0.007 a: 1.011 c:-0.035	0.996	0.0005	3E-07	k: 0.009 a: 0.961 c: 0.006	0.990	0.0013	1.7E-06	k: 0.016 a: 1.004 c: -0.018	0.995	0.001	1E-06
	3	k: 0.013 a: 0.913 c: 0.034	0.973	0.0035	1.2E-05	k: 0.018 a: 0.937 c: 0.033	0.985	0.0022	4.8E-06	k: 0.025 a: 0.980 c: 0.014	0.998	0.0005	3E-07
	4	k: 0.038 a: 0.947 c: 0.046	0.990	0.0016	2.6E-06	k: 0.043 a: 0.957 c: 0.039	0.999	0.0013	1.6E-06	k: 0.042 a: 0.939 c: 0.056	0.984	0.005	2.5E-05

**Table 4:** Parameters and statistical results for the various drying models for blanch-assisted drying at 70°C.

Sample thickness		3 mm				6 mm				9 mm			
Model name	PT (min)	Model constants	*R* ^2^	RMSE	*χ* ^2^	Model constants	*R* ^2^	RMSE	*χ* ^2^	Model constants	*R* ^2^	RMSE	*χ* ^2^

Page	1	k: 0.004 n: 1.309	0.997	0.0006	3.6E-07	k: 0.004 n: 1.191	0.994	0.0009	7.3E-07	k: 0.005 n: 1.141	0.995	0.0007	4.4E-07
	2	k: 0.004 n: 1.322	0.999	0.0004	4E-08	k: 0.004 n: 1.174	0.990	0.0013	1.8E-06	k: 0.010 n: 1.032	0.995	0.0006	4.4E-07
	3	k: 0.024 n: 0.983	0.997	0.0004	3.6E-07	k: 0.045 n: 0.795	0.988	0.0016	2.5E-06	k: 0.009 n: 1.115	0.992	0.0012	1.4E-06

Henderson and Pabies	1	k: 0.016 a: 1.036	0.985	0.0026	6.8E-07	k: 0.009 a: 1.024	0.987	0.0017	3.9E-06	k: 0.011 a: 1.016	0.992	0.0012	1.4E-06
	2	k: 0.017 a: 1.034	0.987	0.0022	4.8E-06	k: 0.00 a: 1.018	0.984	0.0020	4E-07	k: 0.012 a: 1.003	0.995	0.0007	4.4E-07
	3	k: 0.022 a: 0.996	0.997	0.0006	3.6E-07	k: 0.018 a: 0.968	0.980	0.0028	7.8E-06	k: 0.015 a: 1.018	0.989	0.0016	2.5E-06

Logarithm	1	k: 0.013 a: 1.121 c: -0.100	0.993	0.0015	2.3E-06	k: 0.007 a: 1.139 c: -0.141	0.998	0.0003	1.1E-07	k: 0.008 a: 1.118 c: -0.124	0.999	0.0002	4E-07
	2	k: 0.014 a: 1.106 c: 0.085	0.994	0.0013	1.6E-06	k: 0.006 a: 1.193 c: -0.203	0.996	0.0006	3.6E-07	k: 0.014 a: 0.865 c: -0.027	0.934	0.0106	1.1E-04
	3	k: 0.022 a: 1.007 c: -0.013	0.997	0.0005	3E-07	k: 0.019 a: -0.29 c: 0.826	0.980	0.0035	1.2E-05	k: 0.023 a: 0.808 c: 0.035	0.924	0.0143	2.1E-04

**Table 5:** Two-factor three-level factorial RSM design and results of BI, DT, and AA for microwave and blanch-assisted drying of orange-fleshed sweet potato.

Microwave-assisted					Blanch-assisted				
MP (W)	MT (min)	DT (min)	BI (Abs)	AA (mg/g)	BT (min)	ST (mm)	DT (min)	BI (Abs)	AA (mg/g)
697	2	235	0.274	1.8265	1	9	270	0.774	1.7765
541	4	210	0.268	1.7458	3	6	210	0.672	1.6958
541	3	270	0.425	1.8415	2	6	270	0.545	1.7915
697	4	120	0.421	1.4658	3	9	270	0.642	1.4158
541	2	390	0.247	2.1235	1	6	330	0.712	2.0735
541	3	270	0.421	1.8264	2	6	270	0.546	1.7764
541	3	270	0.236	1.873	2	6	270	0.516	1.9046
385	2	450	0.286	2.1654	1	3	210	0.554	2.1154
385	3	330	0.306	1.9548	2	3	210	0.415	1.9048
385	4	270	0.657	1.8462	3	3	210	0.521	1.7962
697	3	175	0.454	1.5647	2	9	270	0.666	1.5147

BI: browning index; DT: drying time; AA: ascorbic acid.

**Table 6:** Model terms, coefficients, and statistical significance for microwave- and blanching-assisted drying on DT, AA, and BI.

Response	*β*_0_	*X*_1_	*X*_2_	*X*_1_ *X*_2_	*X*_1_^2^	*X*_2_^2^	*R* ^2^
DT (MV)	272.89	-86.67	-79.17	16.25	-24.74	22.76	0.9917
*P* value	<0.001^∗^	<0.001^∗^	<0.001^∗∗^	0.043^∗^	0.022^∗^	0.030^∗^	
DT (BL)	270.0	-20.0	30.0	0.00	0.00	-30.0	0.681
*P* value	<0.001^∗^	0.175^∗∗^	0.064^∗∗^	1.00^∗∗^	1.00^∗∗^	0.184^∗∗^	
AA (MV)	2.416	0.0034	-05986	-0.00006	-4E-06	0.0764	0.9948
*P* value	<0.001^∗^	<0.001^∗^	<0.0001^∗^	0.369^∗∗^	0.001^∗^	0.002^∗^	
AA (BL)	2.312	-0.410	0.094	-0.0035	0.063	-0.012	0.9757
*P* value	<0.001^∗^	<0.001^∗^	<0.001^∗^	0.671^∗∗^	0.080^∗∗^	0.012^∗^	
BI (MW)	0.31	-0.003	0.484	-3.6E-04	4E-06	-0.0333	0.5156
*P* value	0.003^∗^	0.757^∗∗^	0.138^∗∗^	0.411^∗∗^	0.307^∗∗^	0.689^∗∗^	
BI (BL)	0.603^∗∗^	-0.42	0.1064	-0.0116	0.1121	-0.0044	0.9095
*P* value	<0.001^∗^	0.073^∗∗^	0.004^∗^	0.176^∗∗^	0.010^∗^	0.214^∗∗^	

^∗^sig at *P* < 0.05; ^∗∗^not sig. at *P* < 0.05; MV: microwave; BL: blanching; X_1_: microwave power/blanching time; X_2_: microwave time/sample thickness; DT is drying time: AA is ascorbic acid content; BI: browning index.

## Data Availability

The data that supports the findings of the study are available within the manuscript.
